# Biomarkers of NRF2 signalling: Current status and future challenges

**DOI:** 10.1016/j.redox.2024.103134

**Published:** 2024-03-30

**Authors:** Christina Morgenstern, Isabel Lastres-Becker, Birsen Can Demirdöğen, Vera Marisa Costa, Andreas Daiber, Roberta Foresti, Roberto Motterlini, Sibel Kalyoncu, Burak I. Arioz, Sermin Genc, Monika Jakubowska, Ioannis P. Trougakos, Aleksandra Piechota-Polanczyk, Michel Mickael, Marlene Santos, Thomas W. Kensler, Antonio Cuadrado, Ian M. Copple

**Affiliations:** a Department of Otorhinolaryngology, Medical University of Vienna, General Hospital of Vienna, Waehringer Guertel 18-20, A-1090, Vienna, Austria; b Institute of Molecular Biosciences, University of Graz, Humboldtstraße 50, A-8010, Graz, Austria; c Department of Biochemistry, School of Medicine, Universidad Autónoma de Madrid (UAM), Spain; d Instituto de Investigación Sanitaria La Paz (IdiPaz), Instituto de Investigaciones Biomédicas “Sols-Morreale” UAM-CSIC, Madrid, Spain; e Centro de Investigación Biomédica en Red de Enfermedades Neurodegenerativas (CIBERNED), Spain; f Department of Biomedical Engineering, TOBB University of Economics and Technology, Ankara, Turkey; g Associate Laboratory i4HB - Institute for Health and Bioeconomy, Faculty of Pharmacy, University of Porto, Porto, Portugal; h UCIBIO - Applied Molecular Biosciences Unit, Laboratory of Toxicology, Department of Biological Sciences, Faculty of Pharmacy, University of Porto, Porto, Portugal; i Department of Cardiology 1, University Medical Center of the Johannes Gutenberg University, Mainz, Germany; j German Center for Cardiovascular Research (DZHK), Partner Site Rhine-Main, Mainz, Germany; k University Paris-Est Créteil, INSERM, IMRB, F-94010, Créteil, France; l Izmir Biomedicine and Genome Center, Izmir, Turkey; m Izmir International Biomedicine and Genome Institute, Dokuz Eylul University, Izmir, Turkey; n Department of Neuroscience, Health Sciences Institute, Dokuz Eylul University, Izmir, Turkey; o Malopolska Centre of Biotechnology, Jagiellonian University, ul. Gronostajowa 7a, 30-387, Krakow, Poland; p Department of Cell Biology and Biophysics, Faculty of Biology, National and Kapodistrian University of Athens, Athens, 15784, Greece; q Department of Cell Cultures and Genomic Analysis, Medical University of Lodz, 90-752, Łódź, Poland; r Department of Experimental Genomics, Institute of Genetics and Animal Biotechnology, Polish Academy of Sciences, Postępu 36A, 05-552, Garbatka, Poland; s REQUIMTE/LAQV, Escola Superior de Saúde, Instituto Politécnico do Porto, Rua Dr. António Bernardino de Almeida, 4200-072, Porto, Portugal; t Translational Research Program, Fred Hutchinson Cancer Center, Seattle, WA, 98109, USA; u Department of Pharmacology & Therapeutics, Institute of Systems, Molecular & Integrative Biology, University of Liverpool, Liverpool, L69 3GE, UK

**Keywords:** NRF2, Transcription factor, Target genes, Biomarker, Oxidative stress response

## Abstract

The cytoprotective transcription factor NRF2 regulates the expression of several hundred genes in mammalian cells and is a promising therapeutic target in a number of diseases associated with oxidative stress and inflammation. Hence, an ability to monitor basal and inducible NRF2 signalling is vital for mechanistic understanding in translational studies. Due to some caveats related to the direct measurement of NRF2 levels, the modulation of NRF2 activity is typically determined by measuring changes in the expression of one or more of its target genes and/or the associated protein products. However, there is a lack of consensus regarding the most relevant set of these genes/proteins that best represents NRF2 activity across cell types and species. We present the findings of a comprehensive literature search that according to stringent criteria identifies GCLC, GCLM, HMOX1, NQO1, SRXN1 and TXNRD1 as a robust panel of markers that are directly regulated by NRF2 in multiple cell and tissue types. We assess the relevance of these markers in clinically accessible biofluids and highlight future challenges in the development and use of NRF2 biomarkers in humans.

## Graphical abstract

**Figure:**
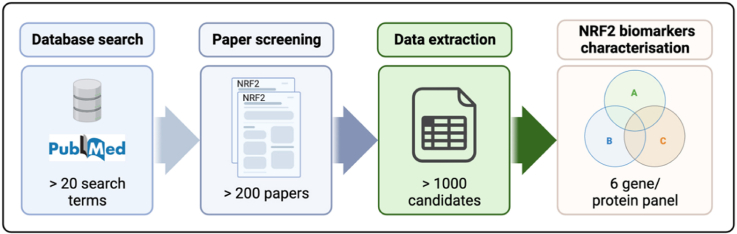

## Abbreviations

AhR: Aryl hydrocarbon receptor
ARE: Antioxidant response element
bZip: Basic region-leucine zipper
ChIP: Chromatin immunoprecipitation
CRISPR: Clustered regularly interspaced short palindromic repeats
CUL3/RBX1: Cullin 3 and RING-box protein 1
ELISA: Enzyme-linked immunosorbent assay
GCLC: Glutamate-cysteine ligase catalytic subunit
GCLM: Glutamate-cysteine ligase modifier subunit
GSK-3β: Glycogen synthase kinase-3β
HMOX1: Heme oxygenase 1
KEAP1: Kelch-like ECH-associated protein 1
MAF: Small musculo aponeurotic fibrosarcoma family
NQO1: NAD(P)H quinone dehydrogenase 1
NRF1: Nuclear factor erythroid 2 like factor 1
NRF2: Nuclear factor erythroid 2 like factor 2
PBMC: Peripheral blood mononuclear cell
ROS: Reactive oxygen species
siRNA: Small interfering RNA
shRNA: Small hairpin RNA
SRXN1: Sulfiredoxin 1
β-TrCP: β-transducin repeat containing E3 ubiquitin-protein ligase
TXNRD1: Thioredoxin reductase 1

## 1 Introduction

**Fig. 1:**
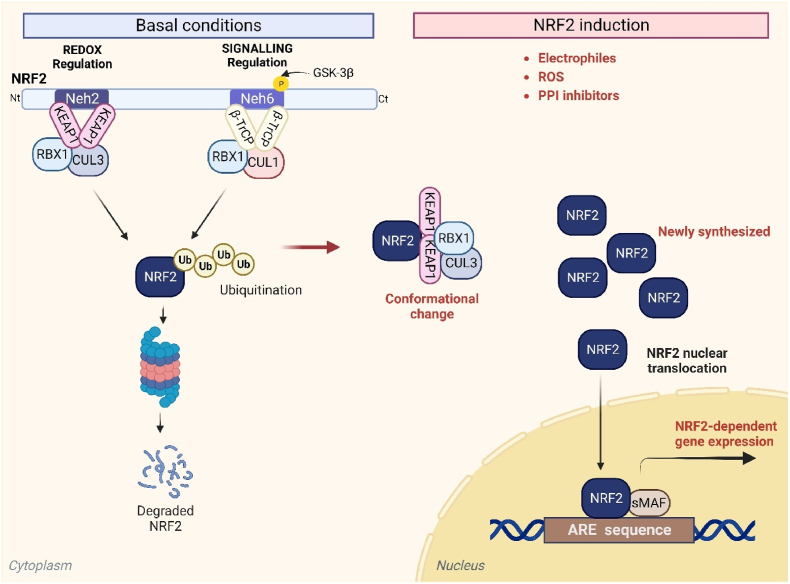
**The NRF2 signalling pathway.** Under basal conditions, a KEAP1/CUL3/RBX1 complex binds to NRF2 and promotes its ubiquitination and subsequent proteasomal degradation. In addition, NRF2 can be regulated via glycogen synthase kinase 3β (GSK-3β) mediated phosphorylation, which promotes ubiquitination of the transcription factor by a β-TrCP/CUL1/RBX1 complex. Upon activation of NRF2 by electrophiles, reactive oxygen species (ROS) and other stimuli, NRF2 evades these repression mechanisms, accumulates in the nucleus, and modulates the expression of several hundred cytoprotective genes.

The basic region-leucine zipper (bZip) transcription factor nuclear factor erythroid 2-like 2 (NRF2), encoded by the *NFE2L2* gene, was first described in connection with its ability to modulate xenobiotic metabolism in cells [1,2]. To date, several hundred genes have been shown to be modulated by this transcription factor. In addition to being an important regulator of xenobiotic metabolism, NRF2 coordinates the expression of genes involved in the antioxidant response, NADPH generation, lipid metabolism, proteasomal degradation/autophagy and mitochondrial biogenesis, among others. The main mechanism that regulates the transcriptional activity of NRF2 is through its binding to the E3 ligase adapter Kelch-like ECH-associated protein 1 (KEAP1), which presents NRF2 for ubiquitination by Cullin 3 and RING-box protein 1 (CUL3/RBX1) and subsequent degradation by the proteasome [3,4] (Fig. 1). Therefore, under homeostatic conditions, NRF2 levels are very low. However, modifications of key cysteine residues in KEAP1 by electrophiles or reactive oxygen species induce conformational changes that impair the ability of KEAP1 to direct NRF2 for degradation. This allows the accumulation of newly synthesized NRF2, which can then translocate to the nucleus, and bind to an enhancer sequence termed the antioxidant response element (ARE) in the promoter regions of NRF2 target genes, together with members of the small musculo aponeurotic fibrosarcoma (MAF) family, and in turn recruit additional components of the transcriptional machinery [5]. An alternative mechanism for NRF2 modulation is through the E3 ligase adapter β-transducin repeat containing E3 ubiquitin-protein ligase (β-TrCP) that presents NRF2 to a CUL1/RBX1 complex, leading to an alternative pathway for ubiquitin-dependent proteasome degradation of NRF2 [6,7] (Fig. 1). Hence, in addition to the numerous pathways that are modulated downstream of NRF2 activation, the transcription factor itself is subject to multiple levels of regulation.

Dysregulation of NRF2 signalling has been linked to several diseases associated with oxidative stress and inflammation, including neurological disorders, liver and other metabolic diseases, and cancer [[8], [9], [10], [11]]. NRF2 has therefore been postulated as a therapeutic target for many of these diseases [12]. To date, a small number of NRF2 activators have been approved for clinical use in different disease settings: 1) Tecfidera®, Vumerity® and Bafiertam®, which have fumarates as the active ingredients and are approved for the treatment of relapsing forms of multiple sclerosis; and 2) Skyclarys®, which contains the triterpenoid omaveloxolone and has been recently approved for the treatment of Friedreich's ataxia. Several other NRF2 activators are in the non-clinical and clinical phases of development. The above approved NRF2 activators, and many others used experimentally, are electrophiles that modify cysteine residues within KEAP1 and inhibit its ability to repress NRF2. However, there are many other cysteine thiols in cellular proteins that could also serve as targets for these molecules [13]. In order to overcome the hypothetical lack of specificity of this approach, recently there has been much interest in the development of protein-protein interaction (PPI) inhibitor-based NRF2 activators designed to target the Kelch domain of KEAP1 and disrupt its interaction with NRF2 [14]. In addition to drug design, an important aspect of pharmaceutical development is the confirmation of target engagement and the ability to monitor pharmacodynamic responses. As a transcription factor, for NRF2 this is typically achieved by quantifying its protein level *per se*, or by measuring the expression level of one or more of the genes it regulates. The latter approach is more reflective of the downstream, functional consequences of NRF2 activation. Yet, due to the pleiotropic effects of NRF2 in different cell types, this approach would be enhanced by defining a consensus set of markers that can be measured across non-clinical and clinical settings, as already achieved for other signalling pathways [15]. The purpose of this article is to establish a guideline for monitoring NRF2 signalling, to support translational research in this exciting scientific area.

## 2 Approaches to the measurement of NRF2 activity

An obvious means of assessing the modulation of NRF2 signalling is to measure the protein abundance of the transcription factor. This has commonly been accomplished through immunoblot analysis using one of the many available antibodies raised against NRF2 [16]. Advantages of this approach include the ability to consider post-translational modifications of NRF2, as well as its subcellular localisation. However, NRF2 protein levels are typically low in cells, particularly in the absence of an activating signal (Fig. 1), and most NRF2 antibodies yield a degree of non-specific binding when used for immunoblotting. In addition, historically there has been some confusion regarding the correct molecular weight at which NRF2 protein should migrate in Tris-glycine sodium dodecyl sulfate polyacrylamide gel electrophoresis [16,17]. Hence, whilst NRF2 immunoblotting can be informative when performed and interpreted with care, it is common practice to use complimentary approaches to confirm the modulation of NRF2 signalling.

Compared with immunoblotting, enzyme-linked immunosorbent assay (ELISA) is a more quantitative approach to determining protein abundance and has been used to measure NRF2 protein levels in a number of experimental contexts. Alternatively, immunohistochemical staining can be used to assess NRF2 abundance semi-quantitatively in fixed tissues or cells. However, these approaches are also dependent on the use of anti-NRF2 antibodies, with potentially greater concerns around specificity compared with immunoblotting due to the inability of ELISA and immunohistochemistry to distinguish between the different targets of an antibody based on molecular weight separation. Ultimately, it is the numerous target genes, or more specifically the associated protein products, that mediate the downstream functional consequences of NRF2 activation. Hence, many investigators report changes in the expression of one or more of these targets (at the mRNA and/or protein level) as a primary means of demonstrating the modulation of NRF2 activity. However, NRF2 is known to regulate the expression of hundreds of genes in mammalian cells, and there is a lack of consensus on which of these targets are the most relevant markers of NRF2 signalling across different experimental contexts. A key consideration is the fundamental definition of a genuine biomarker of NRF2 activity, which is discussed in the following section.

## 3 Defining biomarkers of NRF2 signalling

NRF2 has been reported to influence the expression of several hundred genes across a wide range of *in vitro*, *in vivo,* and clinical settings. In many cases, an observational link has been established by demonstrating that the expression of one or more genes/proteins is altered following the exposure of cultured cells to a pharmacological agent known (or at least hypothesised) to activate NRF2, or the administration of such a compound *in vivo*. Yet, due to the mechanisms of action of these types of NRF2 activators, i.e. electrophilic modification of cysteine residues or disruption of protein complexes, there is potential for some of the associated pharmacological effects to be independent of NRF2. For example, one of the most potent classes of electrophilic NRF2 activators, the cyanoenone triterpenoids (which include Skyclarys®), have been shown to chemically modify cysteine residues in KEAP1 and several hundred other proteins in a concentration-dependent manner [18]. It is also a possibility, although not yet investigated experimentally, that seemingly more specific protein-protein interaction inhibitor-type NRF2 activators could disrupt the interface between the Kelch domain of KEAP1 and its many other binding partners. Hence, mechanistic criteria should be applied in order to categorise a gene/protein as being directly regulated by NRF2, and therefore being a marker of its transcriptional activity.

A direct role for NRF2 in the expression of a number of genes has been evidenced through the use of chromatin immunoprecipitation (ChIP), demonstrating that NRF2 binds to specific ARE regulatory regions within the relevant DNA sequence [19,20]. In other cases, genetic approaches have been used to modulate the basal activity of NRF2. For example, a number of ‘omics analyses have been performed on the tissues of transgenic *Nrf2* knockout mice [[21], [22], [23]]. In addition, siRNA, shRNA, CRISPR and other genetic techniques have been used to knockdown or delete the *NRF2* gene in cultured cells [[24], [25], [26]]. On the other hand, a number of strategies have been used to identify genes/proteins that are sensitive to the activation of NRF2 signalling. Several studies have used pharmacological NRF2 activators in combination with genetic approaches to address the specificity issue highlighted above. For example, by demonstrating that the expression of one or more genes/proteins is significantly altered by a pharmacological agent in wild type but not in *Nrf2* knockout mice [27,28], or in control cells but not in those depleted of NRF2 [29,30], it is possible to define the NRF2-dependence of the observed pharmacological effects. Moreover, selected studies have investigated the consequences of expressing mutated versions of NRF2 that lack an ability to be bound, and therefore repressed, by KEAP1 [31,32]. Genetic manipulation of KEAP1 has also been employed by several investigators [33,34]. However, whole body knockout of *Keap1* in mice causes hyperkeratosis of the oesophagus and the resultant death of weaning pups due to an inability to feed [35]. Instead, hypomorphic *Keap1* mice, in which the gene is partially knocked down but not completely deleted, have been used, along with tissue-specific *Keap1* knockout mice. Given that KEAP1 connects with other signalling pathway regulators beside NRF2, the dependence of the downstream effects of KEAP1 depletion on NRF2 *per se* have been proven by demonstrating a reversal of the outcome upon concomitant depletion or knockout of NRF2 [24,34]. Together, we consider these types of evidence as the most robust demonstration that a gene/protein is directly regulated by NRF2, as opposed to purely observational links reported in studies using pharmacological agents with diverse molecular targets.

## 4 Identification of a robust panel of NRF2 biomarkers

In order to identify a panel of genes/proteins that reflect the modulation of NRF2 signalling across cell types and species, we have conducted an exhaustive literature search based on the following strategy. Firstly, genes/proteins were considered to be candidate markers of NRF2 signalling if they satisfied at least one of the following criteria:

1. Shown to be a direct target of NRF2 using ChIP.
2. Significantly down-regulated in response to inhibition of basal NRF2 activity.
3. Significantly up-regulated in response to activation of NRF2.

This approach identified 1625 genes/proteins as candidate markers of NRF2 signalling (Supplementary Table S1). Our literature search focussed on genes/proteins that exhibit positive regulation by NRF2, i.e. their expression is increased under conditions of NRF2 activation. Notably, some genes/proteins have been shown to be downregulated upon NRF2 activation, yet these were not considered in our literature search given the potential technical difficulties of quantifying a decrease, as opposed to an increase, in the level of a biomarker [36]. For ease of interpretation, we refer to the capitalised human form of biomarkers, even though the literature review covers both genes and proteins, as well as different species. To refine the panel of markers, we focussed only on genes/proteins that satisfied all three of the above criteria, with evidence from at least three separate publications per criteria, spanning 10 or more different cell or tissue types across multiple species. This layer of stringency resulted in a panel of six genes/proteins that we propose as robust markers of NRF2 activity across a range of settings. Specifically, these markers are GCLC, GCLM, HMOX1, NQO1, SRXN1 and TXNRD1 (Table 1). Importantly, we are not suggesting that the remaining 1619 genes/proteins in our original list, or others identified elsewhere, cannot potentially serve as markers of NRF2 signalling. Yet, based on our application of rigorous criteria, we believe that this panel of six genes/proteins represents a consensus set of markers that can be used for the robust analysis of NRF2 modulation in cells and tissues.

**Table 1:** **Summary of evidence for the panel of six genes/proteins representing robust markers of NRF2 signalling across species, based on mechanistic criteria.**^a^Includes studies using *Nrf2* knockout mice and cells in which NRF2 has been knocked down or deleted using siRNA, shRNA or CRISPR. ^b^Includes studies in which pharmacological agents or genetic knockdown/knockout of *Keap1* has been shown to significantly increase the expression of the indicated marker, and reversed by simultaneous *Nrf2* knockout, knockdown or deletion. This category also includes studies using mutated versions of NRF2 that lack the ability to be bound, and therefore repressed, by KEAP1. Blank cells indicate that no relevant studies were identified in the literature search. TXNRD1 has also been shown to be a direct target of NRF2 (ChIP) in bovine species [42]. This table represents a summary of the full literature research (conducted between June and September 2023) for the different criteria that can be found in Supplementary Table S1.

Marker	Direct NRF2 target (ChIP)			Down-regulated upon NRF2 inhibition^a^			Up-regulated upon NRF2 activation^b^		
	Human	Mouse	Rat	Human	Mouse	Rat	Human	Mouse	Rat
GCLC	+	+	+		+		+	+	+
GCLM	+				+		+	+	+
HMOX1	+	+	+	+	+			+	+
NQO1	+	+	+	+	+		+	+	+
SRXN1	+	+		+	+		+	+	+
TXNRD1	+	+		+	+		+	+	+

In the case of HMOX1, it is important to note that the ability of NRF2 to upregulate the expression of this gene has been shown to be dependent on the inhibition of the repressive action of BACH1 [37]. The latter protein essentially blocks the access of NRF2 to AREs upstream of the HMOX1 promoter. For example, the proteasome inhibitor MG132 causes NRF2 accumulation but not BACH1 inhibition, and therefore does not stimulate a marked increase in HMOX1 expression, in contrast to pro-oxidants and other types of NRF2 activators that can inactivate BACH1 and upregulate HMOX1 [37]. Another important consideration in using HMOX1 mRNA as a marker of NRF2 signalling is its distinct kinetics of upregulation and return to baseline level in comparison to many other NRF2 regulated genes. This is exemplified in the response of the mouse liver to a hepatotoxic dose of acetaminophen, whereby *Hmox1* is markedly upregulated early on (peaking at 6 h post-administration) and returns to baseline by 24 h, in contrast to *Gsta1* and *Srxn1* which exhibit a delayed induction that remains near maximal at 24 h [38]. Hence, there may be some instances in which NRF2 is activated and other members of the biomarker panel are upregulated, but HMOX1 is not. This may be influenced by the timing of sample collection for biomarker analysis.

**Fig. 2:**
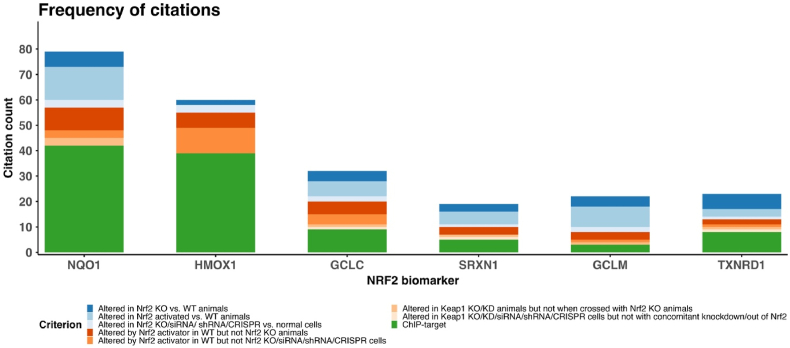
**Frequency of citations for the panel of NRF2 biomarkers.** The stacked bar chart shows the unique count of references falling within the defined criteria for each of the selected genes/proteins. (Diagram was drawn with R 4.2.1 and RStudio version 2023.12.0 + 369 making use of *ggplot2* library.)

In light of the above caveats, a strength of our approach is that it defines a panel of genes/proteins, rather than relying on a single marker of NRF2 activity. Indeed, caution should be used in selecting only one of the six markers, or any other potential single marker, to monitor changes in NRF2 signalling. This is exemplified below for NQO1, which we found to be responsive to NRF2 modulation in the largest number of studies amongst our literature analysis (Fig. 2). The *NQO1* gene encodes NAD(P)H quinone dehydrogenase 1, which contributes to the cellular response to chemical and oxidative stress by catalysing the two-electron reduction and detoxification of quinones [39]. It is perhaps the gene most synonymous with the downstream effects of NRF2 activation and is commonly used as a marker of NRF2 modulation in cells and tissues. However, NQO1 expression is also regulated by the aryl hydrocarbon receptor (AhR) and cannot be upregulated in response to certain chemical inducers in the liver of transgenic *AhR* knockout mice [40,41]. In addition, over 20 single nucleotide polymorphisms in the *NQO1* gene have been reported in humans [39]. Most notably, the NQO1*2 polymorphism leads to a single amino acid substitution that destabilises the encoded protein, resulting in low and almost complete absence of protein expression (as well as enzyme activity) in heterozygous and homozygous individuals, respectively [39]. The frequency of the NQO1*2/*2 homozygous allele ranges from 4 to 34% in different ethnic populations [39]. Therefore, in human studies, the measurement of NQO1 protein could give a misleading reflection of the NRF2 response, in the absence of concomitant patient genotyping. Hence, the above caveats lead us to advise against relying on a single marker and instead recommend the use of a panel of genes/proteins that reflect the modulation of NRF2 signalling.

## 5 Limitations

Whilst this study represents the most comprehensive analysis of candidate markers of NRF2 signalling to date, there are some limitations to our literature-based approach. Firstly, it is important to recognise that we have not undertaken a formal systematic review process, hence some relevant studies could have been omitted from our analysis. Despite this, our literature search identified over 230 publications from which we derived evidence for the construction of the panel of six markers of NRF2 activity. Secondly, we have not performed a meta-analysis of the data from relevant publications to integrate the extent and statistical significance of the reported gene/protein expression changes determined within independent studies. Although this approach could further increase the robustness of our strategy, we believe it is unlikely to materially alter the composition of the panel of NRF2 signalling markers, given the weight of evidence we have found across a range of *in vitro* and *in vivo* settings.

Thirdly, whilst many of our findings are based on unbiased ‘omics analyses, a large proportion of studies only performed targeted quantification of selected genes/proteins known to be associated with NRF2. Indeed, relatively few studies have used ‘omics approaches to assess the consequences of NRF2 modulation in human cells. Hence, there is likely to be some bias in the selection of these markers by investigators and thus the frequency of occurrence in our analysis. This explains why the well-known NRF2 targets NQO1 and HMOX1 were reported to be sensitive to modulation of NRF2 activity in so many publications (Fig. 2). Lastly, our analysis does not take into account any differences between the individual markers in terms of the dynamic range of expression responses to NRF2 activation, nor the potential for distinct kinetics of upregulation and return to baseline levels. The latter phenomenon has been observed in rat liver for different sets of genes associated with NRF2, following administration of therapeutic drugs known to cause hepatotoxicity [43]. These features, and the caveats related to HMOX1 noted above, may influence whether all or a subset of the six markers are significantly altered at a given point within an experiment. To overcome this concern, the expression levels or responses of the six markers could be incorporated into a composite ‘NRF2 activity score’, in a similar manner to a number of recent studies that have used this type of bioinformatic approach to categorise tumours exhibiting high or low NRF2 activity and link this measure to patient outcomes [[44], [45], [46]]. Despite such studies being focussed on human tumours and/or cancer cell lines, in contrast to our literature search which encompassed a range of cell types and species, it is encouraging to note that these ‘NRF2 activity scores’ typically include different combinations of the six markers identified here, along with other genes. This further highlights the universal relevance of our panel of markers across different experimental settings.

## 6 Relevance of the panel of NRF2 markers in clinically accessible biofluids

Given the nature of the available literature, the above panel of NRF2 markers has been identified from studies involving cultured cells or tissues from a range of species. However, there is a need to define a robust means of measuring NRF2 signalling and/or its pharmacological modulation non-invasively, to support clinical trials and other patient studies. Although further experimental work is required in this context, we have cross-referenced the panel of NRF2 markers with gene/protein expression changes reported in human blood or its constituent cells (Table 2), as well as saliva and urine, collected from patients/healthy volunteers following the administration of compounds known to activate NRF2. These sample types represent the most accessible and clinically studied biofluids.

**Table 2:** **Summary of evidence for the pharmacological modulation of the NRF2 marker panel in blood.** All studies involved targeted analysis of selected genes/proteins, rather than unbiased ‘omics analyses. Fold changes in marker gene expression over baseline have been extracted from the respective studies. Bold text indicates the gene/protein was measured and significantly increased. Standard text indicates the gene/protein was measured and not significantly altered. Blank cells indicate that the gene/protein was not measured in the study. *p < 0.05; **p < 0.01; ***p < 0.001; ****p < 0.0001.

Biofluid	PBMCs									Whole blood		Plasma
Pharmacological intervention	Sulforaphane				Omaveloxolone	Bardoxolone methyl	Tecfidera®	Idebenone	Resveratrol	Tecfidera®	Broccoli sprout homogenate	Tin-protoporphyrin
**Reference**	[47]	[48]	[49]	[50]	[51]	[52]	[53]	[54]	[55]	[56]	[57]	[58]
**GCLC**					1.8*			2.3***				
**GCLM**				1.32								
**HMOX1**	3.5**	1.10	0.92				1.28		0.96	0.92	1.53*	3.2***
**NQO1**	3.0**	1.29	0.82	2.42*	1.6*	5.85****	1.3*			2.2*	0.99	1.6*
**SRXN1**					2.4*							
**TXNRD1**					2.4*							

Across the 9 studies we identified in which peripheral blood mononuclear cells (PBMCs) had been isolated from the blood of participants following administration of relevant compounds, there was evidence for modulation of five of the six markers in the panel, albeit not within the same study [[47], [48], [49], [50], [51], [52], [53], [54], [55], [56], [57], [58]]. Clearly, in these clinical settings it is not possible to dissect the role of NRF2 in the response of the panel of markers to the selected pharmacological agents through concomitant genetic inhibition of the transcription factor in circulating cells. Such an approach would be more feasible in translational bridging studies in which cultured PBMCs are exposed to relevant compounds *ex vivo*, for example. The majority of the PBMC studies measured only NQO1 and/or HMOX1. Along with GCLM, these were the only markers for which there was evidence for a lack of modulation by the pharmacological agents (i.e. they were measured but not significantly altered). However, in many cases the markers in the panel simply were not measured, hence at this point it is difficult to draw conclusions about the relevance of the set of markers for monitoring NRF2 activation in freshly isolated PBMCs. We identified two studies in which selected markers from the panel had been analysed in whole blood RNA obtained from patients following administration of NRF2 activating agents [56,57]. Again, NQO1 and HMOX1 were the only members of the panel that were analysed in these studies, with mixed responses reported. We also identified a study in which the protein levels of HMOX1 and, less extensively, NQO1 were shown to be elevated in healthy volunteers and patients with chronic kidney disease following administration of tin-protoporphyrin, which is known to induce mild and acute oxidative stress [58]. Our findings are in agreement with those of Yagishita et al. in their recent analysis of the response of NRF2 target genes and other relevant categories of biomarkers across a large number of clinical studies involving the NRF2 activators Tecfidera®, Bardoxolone methyl, Sulforaphane and Oltipraz [59].

Other clinically accessible biofluids include saliva and urine. In one study, the salivary level of NQO1 protein was increased by 3 to 4 -fold in two healthy subjects following daily consumption of coffee or broccoli [60]. In the aforementioned tin-protoporphyrin study, urinary levels of NQO1 protein were found to increase in a time dependent manner in response to the pharmacological intervention [58]. Other studies have demonstrated that HMOX1 protein can be detected in saliva [61,62] and urine [61,63], although no studies thus far have investigated whether these levels are sensitive to modulation of NRF2 activity in humans. We did not find any reports of the measurement of GCLC, GCLM, SRXN1 or TXNRD1 in saliva or urine. With an expanding number of clinical studies being conducted with NRF2 activators, ‘omics analyses of relevant biofluid samples will become increasingly feasible and help to identify the optimal means of measuring NRF2 signalling non-invasively.

## 7 Metabolite markers of NRF2 signalling

In addition to its established role in regulating genes that possess antioxidant and/or xenobiotic detoxification properties, emerging evidence indicates that NRF2 contributes to reprogramming of cellular metabolism during stress conditions [5]. However, it remains to be determined whether cells and tissues with altered NRF2 activity present a distinct metabolic signature that can reflect modulation of the pathway. Nevertheless, a small number of studies have analysed the metabolomic profiles of cells and organisms either lacking or over-expressing NRF2, raising the possibility of the identification of metabolites that could serve as markers of NRF2 signalling.

Amongst the panel of six NRF2 activity markers defined above, GCLC and GCLM represent the catalytic and modifier subunits, respectively, of the heterodimeric enzyme that serves as the rate-limiting step in the synthesis of glutathione. In a metabolomic analysis of 88 lung cancer cell lines classified based on mutation profiles and associated NRF2 activity status, glutathione was elevated in NRF2-high cell lines, as was l-cysteinylglycine disulphide, an oxidized form of a dipeptide derived from the breakdown of glutathione [64]. One caveat to using glutathione as a metabolite marker of NRF2 signalling is that its levels can be altered by conjugation with electrophiles or oxidants, which could obscure the influence of NRF2 on the initial synthesis of glutathione. In the same lung cancer cell line study, the levels of other metabolites were found to be altered in NRF2-high cells, including glutamate, triglycerides and purine nucleotide products [64]. Modulation of NRF2 signalling in cardiomyocytes has been linked to changes in levels of pentose phosphate pathway products such as ribose-5 phosphate, as well as metabolites such as sorbitol, guanosine diphosphate and choline [65,66]. In addition, an interesting study demonstrated that *Nrf2* deficiency in mice causes similar changes in tissue and circulating metabolite profiles to those exerted by travel to the International Space Station [67]. Affected metabolites included glycine, succinate, triacylglycerol and phosphatidylcholine. Taken together, there is clear evidence that activation or inhibition of NRF2 results in significant changes in cellular metabolism both *in vitro* and *in vivo*. However, most of the metabolic genes and/or pathways downstream of NRF2 are ubiquitous, and thus the levels of associated metabolites are inevitably influenced by a variety of distinct factors not exclusively related to NRF2 activation. In order to address this issue, further work will be required to determine whether a panel of metabolites could be used to infer NRF2 activity, in a similar manner to our proposed approach using NRF2 target genes/proteins.

## 8 Conclusions and future challenges

We have identified a panel of six genes/proteins that are directly regulated by NRF2 across multiple species, representing a set of markers for determining NRF2 activation in cells and tissues. There is evidence that these markers can also reflect changes in NRF2 signalling in accessible biofluids, although more experimental work is required to validate an approach for monitoring NRF2 responses non-invasively in patients. Achievement of this goal will be an important step in enabling the pharmacodynamic effects of NRF2 activating drugs to be tracked in clinical studies. Ultimately, these efforts will be strengthened by collaboration between academic groups and the pharmaceutical companies that are developing NRF2 activators as novel therapeutic agents in a range of disease settings.

An emerging area of interest in the study of NRF2 modulation is the ability of NRF1 and other NF-E2 transcription factor family members to cooperatively regulate the expression of ARE containing genes. Amongst the panel of six NRF2 markers defined here, GCLC, HMOX1, NQO1 and TXNRD1 have been identified in ChIP studies as targets of NRF1, NRF2 and NRF3 [68]. In addition, the six markers have been shown to be downregulated in transgenic mouse livers deficient in NRF2, and to a greater extent in livers deficient in both NRF2 and NRF1, but not in livers deficient in NRF1 [69]. Hence, further work is needed to understand if a panel of markers that is truly NRF2 specific can be identified, in order to support mechanistic research in this area. However, from a practical perspective, given that evidence increasingly suggests that many ‘NRF2 activators’ also modulate NRF1 signalling, it is plausible that these parallel responses and the overlapping downstream consequences both contribute to the resulting therapeutic effects of such compounds. In that sense, a panel of markers that can reflect multiple regulatory inputs may also be valuable.

Some challenges remain regarding the application of NRF2 biomarkers. Our literature search has not distinguished between gene transcripts and proteins when identifying candidate markers of NRF2 signalling. However, this has important implications for the potential use of the panel, particularly in clinical settings. For example, gene transcripts tend to exhibit a larger dynamic range of expression compared to their protein products. On the other hand, proteins are typically better suited to absolute quantitation, as well as inclusion in ‘point-of-care’ diagnostic assays, compared to gene transcripts. The issue of dynamic range is particularly important in the context of establishing a universal ‘normal’ level or range of NRF2 activity in healthy or control individuals. The availability of such a reference point is important if the goal is to develop an approach in which a single, one-off measurement of NRF2 activity is to prove meaningful in a given person. For example, the comparison of biomarker responses between subjects in the placebo and active arms of a clinical trial would be one scenario in which this would be advantageous. However, numerous factors, including age, sex, lifestyle, health status and time of day could influence inter-individual variability in NRF2 signalling. Hence, it will be important to establish the dynamic range and population variability of the panel of markers, at both mRNA and protein levels, as part of any future attempt to develop a clinical NRF2 biomarker strategy. Otherwise, the monitoring of NRF2 activity in an individual will require the comparison of marker levels between two tightly-controlled points, such as before and after the administration of a NRF2 activating therapy, as commonly performed in the relatively small number of clinical studies identified in our literature search (Table 2). This will limit the settings in which NRF2 biomarkers may be valuable, as well as requiring more resources and patient involvement. Another important consideration in the development of NRF2 biomarkers is that, naturally, mRNA demonstrates more rapid upregulation and return to baseline relative to protein, with the latter influenced by the kinetics of ribosomal translation and proteasomal degradation. Indeed, there are marked differences in the protein half-lives of the panel of 6 markers identified here, such as between NQO1 (60 h) and SRXN1 (9.5 h) [70]. As a result, the optimal timing of biomarker measurement may be different depending on the nature of target(s), and it will be important to build this consideration into a further iteration of the panel of NRF2 markers with a view towards clinical application.

Given the relative lack of ‘omics studies performed in human cell models following modulation of NRF2 activity, and evidence that some genes (for example, members of the glutathione S-transferase family [5]) are far more sensitive to NRF2 regulation in mouse cells, an unbiased cross-species comparison of the regulatory roles of NRF2 will be important for ensuring the development of clinical biomarkers is not misdirected by assumptions based primarily on data from other species. Finally, whilst a key translational goal will be to define an optimal panel of markers for assessing NRF2 signalling non-invasively (for example, in blood), it will be important to determine if such an approach is better suited to the broad confirmation of target engagement *in vivo* during drug intervention studies, or if it can also can reflect the modulation of NRF2 in less accessible tissues and therefore serve as a surrogate marker of pharmacodynamic effects at these sites. These questions can be addressed through the use of animal models, human *ex vivo* tissue systems and the increasing number of clinical studies being conducted with NRF2 activating therapies. Tackling these and other challenges will be crucial for maximising our understanding of the role of NRF2 in health and disease, and its true value as a therapeutic target.

## CRediT authorship contribution statement

**Christina Morgenstern:** Formal analysis, Project administration, Writing – original draft, Writing – review & editing. **Isabel Lastres-Becker:** Formal analysis, Writing – original draft, Writing – review & editing. **Birsen Can Demirdöğen:** Formal analysis, Writing – original draft, Writing – review & editing. **Vera Marisa Costa:** Formal analysis, Writing – original draft, Writing – review & editing. **Andreas Daiber:** Formal analysis, Writing – original draft, Writing – review & editing. **Roberta Foresti:** Formal analysis, Writing – original draft, Writing – review & editing. **Roberto Motterlini:** Formal analysis, Writing – original draft, Writing – review & editing. **Sibel Kalyoncu:** Formal analysis, Writing – original draft, Writing – review & editing. **Burak I. Arioz:** Formal analysis, Writing – original draft, Writing – review & editing. **Sermin Genc:** Formal analysis, Writing – original draft, Writing – review & editing. **Monika Jakubowska:** Formal analysis, Writing – original draft, Writing – review & editing. **Ioannis P. Trougakos:** Formal analysis, Writing – original draft, Writing – review & editing. **Aleksandra Piechota-Polanczyk:** Formal analysis, Writing – original draft, Writing – review & editing. **Michel Mickael:** Formal analysis, Writing – original draft, Writing – review & editing. **Marlene Santos:** Formal analysis, Writing – original draft, Writing – review & editing. **Thomas W. Kensler:** Writing – original draft, Writing – review & editing. **Antonio Cuadrado:** Funding acquisition, Writing – review & editing. **Ian M. Copple:** Conceptualization, Formal analysis, Funding acquisition, Writing – original draft, Writing – review & editing.

## Declaration of competing interest

The authors declare the following financial interests/personal relationships which may be considered as potential competing interests: Ian Copple reports article publishing charges was provided by European Cooperation in Science and Technology. Ian Copple reports financial support was provided by the 10.13039/501100000265Medical Research Council. If there are other authors, they declare that they have no known competing financial interests or personal relationships that could have appeared to influence the work reported in this paper.

## Data Availability

No data was used for the research described in the article.
